# Identifying culture as cause: Challenges and opportunities

**DOI:** 10.1017/ehs.2023.35

**Published:** 2024-01-04

**Authors:** Sirio Lonati, Rafael Lalive, Charles Efferson

**Affiliations:** 1 NEOMA Business School – Reims Campus, Reims, France; 2 Faculty of Business and Economics, University of Lausanne, Lausanne, Switzerland

**Keywords:** Causal inference, culture, potential outcomes, treatment effects

## Abstract

Causal inference lies at the core of many scientific endeavours. Yet answering causal questions is challenging, especially when studying culture as a causal force. Against this backdrop, this paper reviews research designs and statistical tools that can be used – together with strong theory and knowledge about the context of study – to identify the causal impact of culture on outcomes of interest. We especially discuss how overlooked strategies in cultural evolutionary studies can allow one to approximate an ideal experiment wherein culture is randomly assigned to individuals or entire groups (instrumental variables, regression discontinuity design, and epidemiological approach). In doing so, we also review the potential outcome framework as a tool to engage in causal reasoning in the cultural evolutionary field.

## Graphical abstract

**Figure:**
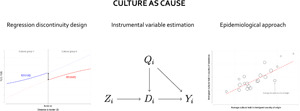

**Media summary:** A review of empirical strategies that can allow estimating the causal effect of culture on outcomes of interest.

## 1. Introduction

Decades of research have documented spectacular behavioural variation across human groups. We eat different foods, speak different languages and believe in different gods. We also have distinct psychologies, diverse economic systems and dissimilar political arrangements. Behavioural variation exists across nation-states, but also sub-national regions, small-scale societies, age groups, and social classes. What causes these differences?

A popular answer is culture, broadly defined as the preferences, values and beliefs each of us learns from parents, peers and older unrelated adults in a group (Richerson & Boyd, 2008). The idea that learned information stored in people's minds can cause behaviour lies at the core of literature on cultural transmission, a theoretical stream of research suggesting that culture can generate behavioural homogeneity within groups and between-group differences that genes alone cannot easily sustain (Henrich & Boyd, 1998). According to this perspective, culture is not just another proximate mechanism that natural selection has deployed to react functionally to the environment, but rather a causal force in its own right. This force can lead groups composed of genetically, demographically and morphologically similar individuals who live in the same environment and access comparable technologies to develop different behaviours.

Yet, while the theory of culture as cause is clear-cut, the empirical reality is considerably messier. The problem is that groups exhibiting different cultural traits can also differ in non-cultural characteristics, like ecology (Lamba & Mace, 2011), institutional environments (North, 1991), demographic factors (Lamba & Mace, 2013) and local genetic adaptations (Fan et al., 2016). As a result, it is impossible to know for sure whether culture or some correlated, unobserved alternative explanations are causing group-level behavioural differences when relying on observational data (cf. Manski, 1993). Adding further complexity, the very composition of different cultural groups is hardly random. It is, thus, unclear whether culture causes group members to behave similarly or whether individuals who are behaviourally similar to begin with choose to join the same group (cf. VanderWeele & An, 2013). In sum, does culture cause group-typical behaviours? Or do group-typical behaviours cause culture? Or are culture and group behaviours both caused by some unobserved factors?

This paper focuses on this major empirical challenge, reviewing notions, research designs and methods that can aid cultural evolutionary researchers in studying culture as cause with observational data. We proceed in three steps. First, we lay some groundwork, presenting the potential outcome framework as a logical–statistical backbone that guides our causal reasoning throughout the paper (Rubin, 1974). Second, we discuss empirical strategies that are commonly used to study culture as cause (e.g. regression where suspected confounders are adjusted for, see, e.g. Major-Smith, 2023), briefly reviewing their limits. Third, we present some empirical strategies that try to approximate an ideal experiment where culture is assigned randomly to individuals or entire groups: instrumental variable estimation, regression discontinuity design and epidemiological approach. As these strategies are rarely used or discussed in cultural evolutionary literature (cf. Bulbulia, 2022; Bulbulia et al., 2021; Muthukrishna et al., 2021), we review their main mechanics and clarify their assumptions in an intuitive way, also discussing some of their potential applications for studying culture as cause.

Note that our paper is a review of different empirical strategies. It is not a statistics cookbook, a how-to manual on specific statistical software, or a review of specific estimators. Rather, we focus mainly on causal identification, broadly intended as the idea that isolating the effect of a variable (culture, in our case) net of alternative explanations always requires invoking some assumptions that are unverifiable from observed data alone. This poses a major difficulty for empiricists, as large samples, Bayesian frameworks or fancy frequentist statistical procedures are *per se* not enough to ensure causal identification. Yet this difficulty does not have to stop researchers from pursuing causal explanations related to culture. On the contrary, these unverifiable identification assumptions can be transparently defended with strong theory, deep knowledge of the empirical context of study, several logical–statistical tools (e.g. directed acyclical graphs, DAGs, see Morgan & Winship, 2015; Pearl, 2010b), and rigorous usage of research designs that can make these assumptions more credible (Angrist & Pischke, 2010; Grosz et al., 2020; Keele, 2015; Lundberg et al., 2021). Such a transparent and principled take on causality is no guarantee of success when studying culture as cause, but it can generate many opportunities for the cumulative evolution of the cultural evolutionary sciences.

## 2. Culture as cause: The challenges

### 2.1. The potential outcome framework

‘Would my headache have stopped if I had taken this pill?’ This type of question is often used in the first pages of introductory readings about causal inference to present the potential outcome framework (e.g. Hernán & Robins, 2020). According to this framework, a causal effect is defined as the difference between the potential outcomes (e.g. headache present or headache absent) that an individual would experience under the two possible treatment conditions (e.g. pill taken or not taken). That is, the causal effect of the treatment for an individual is the difference between the two ‘parallel realities’ that could in principle have come into being for that individual (i.e. felt headache had the individual taken the pill*−*felt headache had the individual *not* taken the pill; Schwartz et al., 2012).

As this causal effect is defined at the individual level, it can never be observed directly. The problem is that any given individual either takes the pill or does not take it and cannot be observed under both treatments. As a result, only one potential outcome is observed for each individual, while the other outcome remains counterfactual (i.e. contrary to fact) and is never observed. This is the ‘fundamental problem of causal inference’ (Holland, 1986). Note how this problem is not an *estimation* issue, but rather an *identification* one. That is, no matter how large our sample or how sophisticated the statistical techniques we use, there is simply no way to identify (i.e. write) the unobservable individual causal effect of the pill as a function of the observable data alone (see Table 1).

**Table 1.** The fundamental problem of causal inference

Unit	Treatment	Observed headache	Potential headache w/ pill	Potential headache w/o pill	Individual causal effect
1	No pill	1	1	?	1–?
2	No pill	0	0	?	0–?
3	No pill	1	1	?	1–?
4	Pill	0	?	0	?–0
5	Pill	0	?	0	?–0
6	Pill	1	?	1	?–1

How can we ever solve this conundrum? While individual causal effects are inherently unknowable, their *average* can in principle be identified. That is, if researchers observe multiple individuals, some of whom took the pill and some of whom did *not* take the pill, it is possible to use the distribution of the outcomes of the untreated individuals to approximate what would have happened to the treated ones had they taken no pill. For instance, researchers can calculate the average treatment effect as the difference between the population average of the felt headache among the individuals who took the pill and the population average of the felt headache among the individuals who *did not* take the pill (other types of aggregated causal effects can also be calculated, see, e.g. Hernán & Robins, 2020).

Observing multiple individuals is, however, not sufficient to identify the average treatment effect. Identification requires the observed outcomes of the untreated units to approximate well (i.e. act as ‘plausible substitutes’ for) the unobserved outcomes that the treated ones would have experienced had they not been treated, and vice versa. Yet, if treated and untreated individuals are different to start with, this approximation will be misleading. For instance, assume that only the individuals with a headache decide to take the pill and that the pill can only cure mild forms of migraine. In this case, a naive comparison between the average felt headache of treated and untreated individuals might lead to a paradoxical – and most certainly incorrect – conclusion. All individuals who did not take the pill are feeling just fine, while some of the individuals who took the pill have a headache. Thus, pills against headache must give a migraine (Angrist & Pischke, 2009)!

In most academic fields, randomised experiments are the gold standard to ensure that the treated individuals are very similar – treatment status aside – to the untreated ones. In an experiment, individuals are assigned to take the treatment by chance alone. As a result, individuals who end up taking the treatment will tend to be similar in expectation to the ones who do not. In potential outcome terms, randomisation ensures that individuals’ potential outcomes do not depend on the treatment assignment so that untreated individuals’ outcomes can serve as good proxies for the counterfactuals of the treated individuals (and vice versa, see Hernán & Robins, 2020).

Technical box 1: Potential outcome framework and identification

Following Rubin (1974), consider a large population of units indexed with the subscript *i*. These units can be exposed to a treatment *D_i_* (e.g. taking a pill), which can either be present (*D_i_* = 1) or absent (*D_i_* = 0). Each unit can have two potential outcomes, *Y_i_*(1) and *Y_i_*(0), corresponding to the outcome the *i*th unit would have experienced had it respectively been exposed to the treatment or not. For each unit, only one reality obtains, and the researchers only observe the realised outcome, *Y_i_* (e.g. headache level):
1
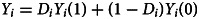

Equation (1) (which implicitly assumes consistency and no-interference, see Section 2.2) allows us to define the causal effect of the treatment for the *i*th unit as
2
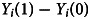

This individual causal effect is fundamentally unidentifiable because we cannot observe the same unit under different values of the treatment *D_i_* (Holland, 1986). We can, however, sometimes learn at least its average, the average treatment effect (ATE):
3
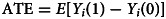

The ultimate objective of causal inference is to identify ATE (or alternative measures of causal effects), that is, to calculate a quantity defined in terms of potential outcomes with observed quantities, like *E*[*Y_i_|D_i_* = 1] (i.e. the population average of the observed headache for individuals who took the pill) and *E*[*Y_i_|D_i_* = 0] (i.e. the population average of the observed headache for individuals who did not take the pill).

### 2.2. From pills to culture: The consistency condition

The pill–headache allegory illuminates some key notions in causal inference, like the definition of a causal effect, the fundamental problem of causal inference and the special status of randomised control trials. Yet this toy example might seem to lose its grip once we turn our attention to more complex treatments, like culture. Unlike a pill, culture can hardly be randomly assigned to individuals or entire groups by some researchers. Rather, culture is an intangible ‘thing’ that stems from social learning dynamics and thus requires some time to take place. As a result, researchers cannot just force individuals to deeply internalise some social information at random and observe how the effect of this information unfolds. Yet if culture is so different from the medical treatment of our toy example, does it make any sense to rely on the potential outcome framework to think about culture as cause?

We believe not only that using the potential outcome framework to study culture as cause is possible, but also that doing so allows for great conceptual discipline – something crucial when tackling causal questions. To see why this is the case, we need to discuss a condition that is at the core of the potential outcome framework: consistency (Hernán & Robins, 2020). Consistency requires that, for any given level of the treatment a unit is exposed to, researchers observe the potential outcome for that treatment (consistency has, thus, been regarded either as an assumption or as a self-evident axiom needed to define potential outcomes by different researchers, see, e.g. Keele, 2015; Pearl, 2010a; Rehkopf et al., 2016). For instance, consistency means that whenever a unit takes the pill and develops a certain observed headache level (i.e. either having or not having a headache), then its potential outcome under the treatment ‘taking the pill’ is also the observed or experienced headache level.

In the toy pill–headache example, the consistency condition might seem nothing more than a triviality. Yet once we focus on more complex treatments, consistency starts to show its teeth. Consider for instance another commonly used remedy against headache: resting. Resting is a considerably broader and less well-defined treatment compared to taking a pill, begging a simple question: what does ‘resting’ (and, conversely, ‘not resting’) mean? Perhaps, resting involves closing one's eyes for a couple of minutes, or maybe staying in bed for a couple of days. Yet here is where a violation of consistency can emerge. Consistency implies that different sub-components or versions of the treatment have the same effect on the outcome (Rehkopf et al., 2016). Yet resting for a couple of minutes or a couple of days will probably have different effects on felt headache. As a result, if we simply observe some people who rested for a vaguely defined amount of time and compare them with individuals who did not rest in a similarly unspecified way, we lose the link between observed and potential outcomes. Are we comparing individuals who stayed in bed for days to individuals who worked for 12 hours in a row on a tight deadline? Or individuals who rested for a couple of minutes during a long day of work with individuals who worked casually for a couple of hours? As the very meaning of ‘resting’ is unclear, its causal effect cannot be interpreted unambiguously.

Here is a powerful insight provided by the potential outcome framework. Whenever our treatment of interest is vaguely defined, consistency can be violated, leading to an inherent vagueness of the causal question of interest (Hernán & Robins, 2020). Major consistency violations can arise especially when researchers focus on complex and multidimensional treatments (e.g. culture) that do not correspond to an actual intervention that could be really conducted in the field (e.g. taking a pill).

Thus, if we want to make progress in our causal quest about culture, we first need a precise definition and operationalisation of culture (Janes, 2006). Luckily, most cultural evolutionary scholars agree on such a definition: culture is information – that is, preferences, beliefs, knowledge and norms – that is socially learned and transmitted (see Mesoudi, 2020; Richerson & Boyd, 2008). Thus, culture is not so different from a medical treatment, at least conceptually. At its core, culture is just a single piece of information that individuals have either learned or not learned. As such, cultural evolutionary researchers can certainly ask counterfactual questions about the potential effects of the presence or absence of this single piece of information, having some hope that the consistency condition could be satisfied. For instance, researchers could ask whether a specific cultural trait (e.g. the presence of ‘big gods’, that is, powerful moral, and omniscient gods in a society; Norenzayan, 2013) causes a specific outcome (e.g. cooperation across societies measured with a behavioural game) in a specific population (e.g. all individuals in the world) by asking a simple question at the individual level, like ‘would an individual living in a society with big gods be as cooperative if she had been from a society without big gods?’ Researchers could also ask a similar question at the group level, for instance, ‘would a society where a belief in big gods is present be as cooperative if it had had no big gods?’

Note, however, that a precise definition of culture does not guarantee that consistency is automatically satisfied when studying culture. Because absolute clarity about the definition and operationalisation of culture is hard to attain, we shall be careful when pushing the equivalence between culture and the pill of our toy example too far. The main issue is that it is often hard to pin down the exact piece of social information that makes for the treatment ‘culture’. For instance, if one asks whether societal beliefs regarding the presence of big gods cause cooperation, consistency violations might emerge because big gods might not represent a single bit of transmitted information, but rather a constellation of societal ideals, norms or beliefs. In turn, each specific facet of this constellation might have different effects on cooperation. Similarly, consistency violations might emerge if learning to believe in big gods from different sources (e.g. parents, peers, older unrelated individuals) has different effects on cooperation.

Minimal vagueness in the definition and operationalisation of culture can, in principle, lead to consistency violations. Yet whether such breaches are enough to be a serious concern depends entirely on the research question and on the empirical setting at hand (for a discussion, see Keele, 2015; Morgan & Winship, 2015). For instance, in our big gods example, different degrees or types of moralising religions might exist (Fitouchi et al., 2023). As such, the treatment ‘big gods’ might fail consistency if researchers are interested in a specific facet of moralising religions. Conversely, if researchers are interested in a more aggregate – though less precise – cultural norm linked to moralising religions, the treatment ‘big gods’ might be precise enough. Note that even a treatment as simple as the one of our toy example (i.e. taking a pill) might fail consistency. For instance, whether one takes the pill willingly or not, one takes the pill in the morning or in the evening, or takes the pill before or after lunch might have very different effects, leading to potential consistency issues. It is, thus, up to the researchers to decide whether a treatment is sufficiently well defined for the purpose at hand or if makes sense to re-frame the entire causal questions (Hernán & Robins, 2020). For this reason, throughout the remainder of this paper, we will assume that consistency holds, unless specified otherwise (note that the identification and interpretation of causal effects under failures of consistency is a novel and active area of research, see, e.g. VanderWeele & Hernán, 2013).

Note, finally, that consistency is related to another condition – no interference. No interference means that the treatment value of any unit does not affect the other units’ potential outcomes (for an intuitive discussion, see, e.g. Keele, 2015, Section 2.1). This assumption is probably easily met in our pill–headache toy example, as the pill intake of an individual is unlikely to have an effect on other individuals’ headaches. Yet no-interference might be violated when studying individuals’ interactions with each other, as often happens when studying culture. When this is the case, the potential outcomes are also not well defined, to the extent that they do not only depend on the focal individual's treatment status but also on all other individuals’ treatment statuses. Violations of no-interference can thus be as problematic as violations of consistency. Different from consistency, however, we see no interference as mainly an empirical issue. While we assume throughout our paper that no interference holds, we discuss it more in detail in Section 4.1.

### 2.3. From randomisation to conditional randomisation: Unconfoundedness and positivity

Consistency and no-interference are necessary conditions to proceed with our causal quest about culture. Yet a major problem remains: culture can rarely – if ever – be randomised. Rather, cultural evolutionary researchers can often just observe units that express different cultural traits and try to infer a causal nexus between culture and an outcome of interest. However, if culture cannot be randomised, are we not back to square one? Not necessarily. Identification of average treatment effects is still possible in observational studies, even though it becomes significantly more difficult, requiring two additional assumptions: unconfoundedness and positivity (Hernán & Robins, 2020, see also Technical box 2).

To better understand these two assumptions, let us add some nuances to the big gods → cooperation example we already introduced. Specifically, let us imagine some researchers who have collected data on cooperation across different societies and have found an association between cooperation rates and the presence vs. absence of beliefs concerning big gods. Let us also assume that researchers have noted that societies with big gods are on average more complex (e.g. more market-integrated, with more jurisdictional layers) than societies without big gods (cf. Henrich et al., 2010a; Purzycki et al., 2023). The key question we ask is: in this setting, is the observed association between beliefs in big gods and cooperation at the societal level causally interpretable?

In this simple example, big gods are clearly neither randomised to societies by the researchers nor *as-if* randomised by nature. Had randomisation occurred, we would not observe any meaningful difference in societal complexity (or other observable characteristics) across societies with and without big gods (Hernán & Robins, 2020). Yet, if societal complexity is really *the sole factor* causing cooperation that is also unequally represented across societies with and without big gods, then researchers can still identify the causal effects of big gods. In this case, we say that our treatment (i.e. big gods) is unconfounded when controlling for the observed covariate (i.e. societal complexity) or, simply, that unconfoundedness holds. Note, unconfoundedness is also called conditional exchangeability, conditional independence, weak independence, ignorable treatment assignment, selection on observables and no omitted variables (see, e.g. Abadie & Cattaneo, 2018; Angrist & Pischke, 2009; Hernán & Robins, 2020; Imbens, 2004; Rosenbaum & Rubin, 1983).

Unconfoundedness is well understood in cultural evolutionary studies (Major-Smith, 2023), but it is not enough to make causal claims in observational studies. Rather, researchers need to also invoke positivity. Positivity implies that, for each level of the covariate, there is a good mix of treated and untreated units. In our example, this means that we can observe at least some societies that either have or do not have big gods for each possible value taken by the variable societal complexity (Rosenbaum & Rubin, 1983). Intuitively, while unconfoundedness ensures that the presence of big gods can be regarded as assigned randomly conditionally on societal complexity, positivity means that, for each level of societal complexity, the (as-if) randomisation of big gods actually took place. That is, if both conditions hold, researchers can see their data as a collection of many randomised experiments where, for each level of societal complexity, the presence or absence of big gods is as good as randomly assigned (cf. Hernán & Robins, 2020).

Technical box 2: Randomisation and selection on observables

Relying on the notation and setting of Technical box 1 and assuming that consistency and no-interference hold, let us consider a treatment *D_i_* and an outcome *Y_i_*. When *D_i_* is randomised, the following property holds (e.g. Abadie & Cattaneo, 2018):
4
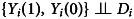

Equation (4) means that the treatment is assigned independently of the potential outcome values for all units *i*. Randomisation allows identification of the ATE, as for any level *d ∈ {*0, 1*}* of the treatment *D_i_*:
5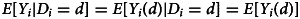 where the first equality stems from consistency and no-interference (as implied by Equation 1) and the second equality stems from Equation (4). As a result, *ATE* = *E*[*Y_i_|D_i_* = 1] *− E*[*Y_i_|D_i_* = 0]. That is, ATE is identified by the typical contrast between the average outcomes in two different (usually experimental) groups that are found in many empirical papers.

Consider now a situation where the same treatment *D_i_* is not randomised, but the researchers observe a covariate (or group of covariates), *X_i_*. In this case, Equation (5) is not ensured to hold. However, we say that the treatment is strongly ignorable if, for every level *x* of the covariate(s), the following assumption holds (Rosenbaum & Rubin, 1983):
6
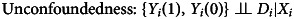

7
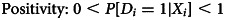

Under these assumptions, we can identify a conditional version of ATE knowing that
8
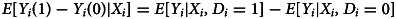

### 2.4. Identification threats in observational studies

In theory, unconfoundedness and positivity are remarkable properties, allowing researchers to make causal claims without randomisation. However, there are at least three reasons why these two assumptions might be often violated in practice: omitted common causes, conditioned common effects and random positivity violations.

**Figure 1.**
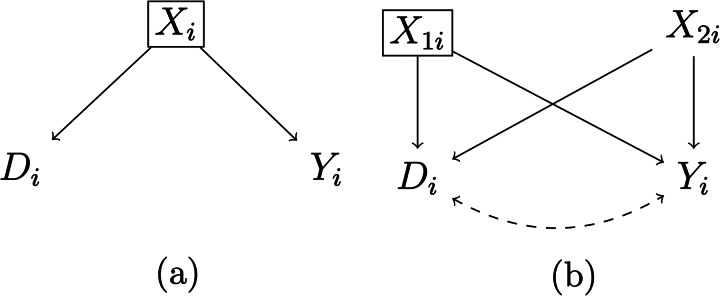
Selection on observables and omitted common causes. *Note:* A box around a variable means that this variable is conditioned on in the analysis; a dashed arrow represents a spurious relationship. (a) A case where conditioning is enough to correctly identify the null causal effect of *D_i_*; (b) a conditioning strategy that does not completely solve issues of unobserved common causes, because only the common cause *X*_1_*_i_* is observed, while the common cause *X*_2_*_i_* is unobserved.

*Omitted common causes*. Unconfoundedness ultimately requires observing and modelling all covariates that cause both the cultural trait and the outcome of interest. As such, it is a heroic assumption that can never be tested. For instance, in the big gods–cooperation example, societies with big gods might not only be more complex, but also have other demographic, socio-economic, and institutional differences compared to societies without big gods. However, given that researchers have only measured societal complexity, there is simply no way to know if there are other predictors of the outcome that are also unequally distributed across treated and untreated units. If unobserved, these omitted predictors (usually called omitted common causes, confounders, or omitted variables, see, e.g. Cinelli et al., 2022) can engender spurious relations between treatment and outcome (see Figure 1a).

Note, however, that it would be unfair to portray the issue of omitted common causes as a ‘yes vs. no’ problem. While a correct point identification of the average treatment effect requires modelling all common causes of treatment and outcome, adjusting for some common causes can at least decrease bias (see Figure 1b). For instance, if one adjusts for societal complexity but fears that the size of the society might be another omitted common cause, a part of the bias driven by societal size will be accounted for because larger societies are also more complex. Moreover, cultural evolutionary scholars can rely on several tools to guide their reasoning about omitted common causes. Most notably, DAGs are a common logical–statistical tool to guide covariate selection (for an example in cultural evolutionary studies, see Major-Smith, 2023). Cultural evolutionary researchers can also rely on sensitivity analyses to assess the fragility of their results to confounding (e.g. Cinelli & Hazlett, 2020). Yet despite their usefulness, these tools do not change the nature of the omitted common cause issue, which remains an ultimately untestable problem.

**Figure 2.**
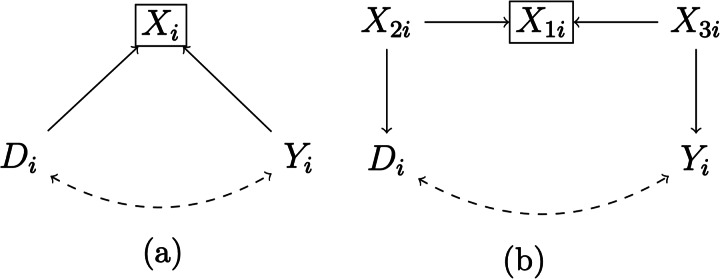
Conditioning on a common effect and M-bias. *Note:* A box around a variable means that this variable is conditioned on in the analysis; a dashed arrow represents a spurious relationship. (a) Conditioning on the common effect *X_i_* engenders a spurious relation between the treatment *D_i_* and the outcome *Y_i_*. (b) Conditioning on the variable *X*_1_*_i_* engenders a spurious relation between the treatment *D_i_* and the outcome *Y_i_*.

*Conditioned common effects*. Given the dangers of omitting common causes, conditioning on (i.e. adjusting for) as many variables as possible in a regression analysis or similar could be seen as a sensible approach. Yet even this strategy might do more harm than good, because violations of unconfoundedness can also emerge when conditioning on variables that should be left out of the analysis. This problem emerges most clearly when researchers explicitly condition for common effects, that is, variables that are caused by both outcome and predictor of interest (see Figure 2a). This problem is related to an issue that might be familiar to evolutionary scholars – colliders (Cinelli et al., 2022; Elwert & Winship, 2014; Hernán et al., 2004).

In principle, issues related to conditioning on common effects are easy to avoid, requiring researchers to adjust only for covariates that are determined *before* the cultural trait of interest (cf. Montgomery et al., 2018; Rosenbaum, 1984). Yet this golden rule can be difficult to implement in practice, as there is often considerable doubt about when a certain cultural trait emerged. For instance, in our big gods–cooperation example, should a researcher adjust for societal complexity? If societal complexity predates big gods, one might want to condition on it to limit unconfoundedness violations owing to omitted common causes (e.g. a more complex society might be more prosocial and exhibit big gods to begin with). However, if societal complexity postdates big gods and is also an outcome of cooperation, then conditioning on it could lead to collider bias. Moreover, even conditioning on an antecedent of the treatment could lead to collider bias if such antecedent is caused by unobserved variables that cause, respectively, treatment and outcome (i.e. the so-called ‘M-bias’ structure, see Figure 2b; see also Greenland, 2003).

Note how a similar problem can also emerge without conditioning explicitly on a common effect, but when doing so implicitly in the sampling stage. This issue – often called selection bias or endogenous selection bias – encompasses myriad cases, ranging from outright selection on the dependent variable to more intricate cases (for details, see, e.g. Elwert & Winship, 2014). The key idea, however, is that selection bias is different from issues of generalisability stemming from non-representative samples. Selection bias relates to the fact a sample selected based on a common effect will lead to a violation of unconfoundedness, thus causing an identification/internal validity problem. For instance, if a higher presence of big gods and a higher rate of cooperation both cause societies to be observed by researchers, then the observed relationship between the two might be spurious. On the contrary, non-representative samples *per se* do not imply such a problem. For instance, obtaining a non-representative sample in a survey or sampling only WEIRD subjects (Western, Educated, Industrialised, Rich, Democratic) clearly reduces the generalisability of some empirical results (Henrich et al., 2010b). Yet non-representative samples do not imply that relationships found among the selected sample are causally uninterpretable *per se*.

*Random positivity violations*. Conditioning on many covariates might also lead to a final issue – random positivity violations (note, structural positivity violations also exist, but they are more of a conceptual issue that we do not cover in this paper, see Petersen et al., 2012; Westreich & Cole, 2010). Random positivity violations arise when researchers happen by chance to observe only treated or untreated units for a given level of the covariates that are supposed to ‘de-confound’ the treatment–outcome relationship of interest. Note that this issue might be relatively unlikely in our big gods–cooperation example, where we assumed that only societal complexity was needed to satisfy unconfoundedness. However, positivity requires researchers to observe a good mix of treated and untreated units *for each combination of levels of the covariates*. Thus, positivity violations become much more likely (D'Amour et al., 2021) and hard to diagnose if researchers start to condition on multiple covariates, if the covariates have many levels, or if the covariates are continuous variables (although positivity violations can be, at least partially, detected, especially if one uses methods based on the propensity score, Rosenbaum & Rubin, 1983). This can lead to an explosion of the number of conditionally randomised experiments we need to assume if we want to leverage unconfoundedness. For instance, with one covariate having two levels, we need to have a good mix of societies with and without big gods in 2^1^ = two cells, with two binary covariates 2^2^ = four cells, with three binary covariates 2^3^ = eight cells, with four binary covariates 2^4^ = 16 cells, etc.

Finally, note that when random non-positivity is present, researchers can still identify aggregate causal effects. However, researchers need to rely on parametric extrapolation as a ‘substitute’ for positivity (for details, see Hernán & Robins, 2020). This solution is in principle straightforward, but it requires additional parametric assumptions that might be hard to defend (i.e. correctly specified model).

## 3. Culture as cause: The opportunities

The reader might feel without options at this point. Randomisation is often out of the question when studying culture as cause. Yet causal identification with observational data requires strong and partially untestable assumptions. Almost paradoxically, these assumptions can be violated when adjusting for both too few and for too many variables. So, how can we make further progress when studying culture as cause? In this section, we discuss the possibility that researchers might sometimes do better than just adjusting for a handful of observed covariates and hoping for the best. Rather, researchers can actively look for naturally occurring events, characteristics and settings that can *approximate by design* an ideal randomised control trial where culture is assigned haphazardly to individuals or entire groups. Specifically, we suggest that cultural evolutionary researchers can take advantage of the three empirical strategies we review next: instrumental variables estimation, spatial regression discontinuity design (and related approaches) and ‘epidemiological approaches’.

### 3.1. From randomised experiments to randomised encouragement designs: Instrumental variable estimation

In this section, we review instrumental variable estimation. Instrumental variable estimation is a powerful strategy, which allows making causal claims about culture even when unconfoundedness does not hold. To unleash its power, however, this empirical strategy relies on an alternative set of identification assumptions.

#### 3.1.1. Identification assumptions

To understand the logic behind instrumental variables, let us consider our initial toy example about pills and headaches. However, let us now assume that researchers cannot randomise individuals to actually ingest the pill, but that they can only encourage subjects to do so (i.e. a ‘randomised encouragement design’; Holland, 1988; Keele, 2015). For instance, researchers might send a daily reminder to treated subjects to take the pill (cf. Hirano et al., 2000). In this setting, it is ultimately each subject's decision whether or not to take the medical treatment, yet the encouragement they receive acts as a random push to take the pill. As such, the pill → headache relationship is confounded, but both the encouragement → pill and the encouragement → headache ones are not. Thus, if the researchers are ready to assume that the encouragement affects the outcome of the study (e.g. the felt headache of a person) only via the intake of the treatment (i.e. actually ingesting the pill), then the random encouragement can be seen as a prototypical instrumental variable, also called ‘instrument’.

**Figure 3.**
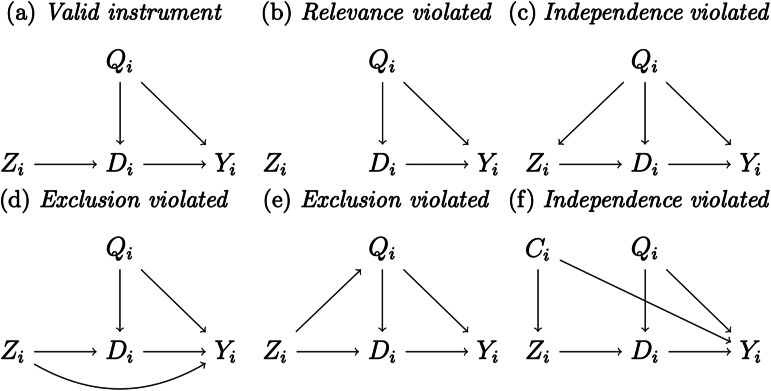
Instrumental variables (see Huntington-Klein, 2021). *Note:* All panels display the relationship between a valid or invalid instrument *Z_i_*, a cultural trait *D_i_*, an outcome *Y_i_* and two potentially omitted common causes, *Q_i_* and *C_i_*.

This example lacks cultural or evolutionary subtlety, yet hints at the power of instrumental variable estimation for cultural evolutionary scholars. The intuition is as follows. While it is usually impossible to randomise culture directly, it might be feasible to find a (conditionally) random variable that causes culture. This variable – the instrument – can be thought of as the initiator of a causal chain that ‘de-confounds’ the effect of a cultural trait of interest (Angrist & Pischke, 2009). To produce such a powerful result, instruments need to satisfy three assumptions (Angrist & Pischke, 2014, see Figure 3 for an intuitive representation):

1. *Relevance*. Relevance means that the instrument should be associated with the treatment (e.g. the cultural trait of interest).
2. *Independence*. Independence is similar to unconfoundedness and requires the instrument (but not the treatment) to be at least as good as random.
3. *Exclusion*. Exclusion means that the instrument should affect the outcome solely via the treatment (e.g. the cultural trait of interest) and not via any other unmeasured variable. Readers familiar with statistical mediation literature might intuitively think about exclusion as the assumption that requires the treatment to mediate fully (rather than partially) the instrument–outcome relationship (cf. Baron & Kenny, 1986).

Of these three assumptions, only relevance can be directly tested by measuring the empirical association between instrument and treatment (for details, see, e.g. Andrews et al., 2019). Independence – like unconfoundedness – is untestable but can be assumed as valid when the instrument is randomised (Angrist & Pischke, 2009). The exclusion assumption is also untestable, yet it is not assured to hold even in a randomised experiment, as the randomised instrument might affect the outcome through a channel different from the (endogenous) treatment.

Note that these assumptions are enough to characterise an instrumental variable in a scenario with homogeneous effects, a simplifying assumption describing a world where the effect of the cultural trait on the outcome of interest is identical for all units in the population. However, an additional assumption called ‘monotonicity’ (or ‘no-defiers’) is usually required when focusing on a more general heterogeneous effects scenario (for details, see Angrist et al., 1996, or Technical box 3). Monotonicity boils down to assuming that no unit does the contrary of what its instrument level would imply, also allowing us to clarify the interpretation of the instrumental variable parameter as a local average treatment effect, that is, the effect of the treatment among the units that respond to the instrument.

#### 3.1.2. Estimation: Basic notions

The most intuitive way to think about instrumental variable estimation in the homogeneous effect case is via an estimation procedure called ‘two-stage least squares’ (Angrist & Pischke, 2014). Two-stage least squares relies quite literally on the causal chain implied by an instrumental variable and estimates two different equations. The first stage usually takes this form:
9
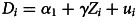
and is estimated via an ordinary least squares estimator, wherein *D_i_* is the cultural trait of interest explained by the instrument *Z_i_*, by a constant term, *α*_1_, and by a disturbance, *u_i_*. Intuitively, the value of *D_i_* predicted by this regression equation can be thought of as the unconfounded variation in the cultural trait that is driven only by those units who responded to the instrument. The second stage requires regressing the outcome *Y_i_* on the values of *D_i_* predicted by Equation (9), thus obtaining an estimate of the effect of culture ‘purged’ of confounding if the identifying assumptions of instrumental variable estimation hold.

#### 3.1.3. Where to find instruments and how to argue for their validity?

In the context of culture as cause, a valid instrument is a variable that is (as good as) randomly assigned, but that strongly predicts culture and causes the outcome of interest only through its effect on culture. This is a demanding and rather distinctive set of characteristics, making it difficult to even think about an instrumental variable in many applied scenarios. Indeed, as succinctly put by Cunningham (2021), ‘[g]ood instruments should feel weird’ (chapter 7.2.2). So, where to look for such surrogate random assignment to treatment when studying culture as a cause? Aside from relying on the physical random assignment typical of actual experiments, researchers might look for as-if random variation that emerges naturally in the field. We now discuss some examples related to culture that clarify this logic.

*The effect of collectivism on economic development.* In a series of studies, Gorodnichenko and Roland examine one of the most studied psychological differences across societies: collectivism vs. individualism (Gorodnichenko & Roland, 2011a, 2011b, 2017, 2021). This psychological continuum measures whether members of society attribute greater importance to individual goals and personal freedom (i.e. individualism) or group goals and conformity (i.e. collectivism, Hofstede, 2001). At the country level, the collectivism–individualism continuum shows a remarkable correlation with economic development, but is this relationship causally interpretable or is it just driven by unobserved confounding?

Technical box 3: Instrumental variable estimation

Following the notation of Angrist and Pischke (2009), consider an instrument, *Z_i_*, that can take two values, *z ∈ {*0, 1*}*, and that affects a cultural trait, *D_i_*, which can also take two values *d ∈ {*0, 1*}*. To formalise the logic of instrumental variable estimation, we need to augment the potential outcome notation. Specifically, we can think of the cultural trait as a potential outcome in terms of the instrument, *D_i_*(*Z_i_*). Similarly, we can think about the potential outcome for our outcome of interest as dependent both on the cultural trait and on the instrument, *Y_i_*(*D_i_*(*Z_i_*), *Z_i_*). Assuming that consistency and no-interference hold, we can define the instrument *Z_i_* as a variable that satisfies (Angrist et al., 1996):

- Independence: *{Y_i_*(*D_i_*(1), 1), *Y_i_*(*D_i_*(0), 0), *D_i_*(1), *D_i_*(0)} 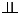 *Z_i_*.
- Exclusion: *Y_i_*(*d*, 0) = *Y_i_*(*d,* 1) for *d ∈ {*0, 1*}*.
- Relevance: *E*[*D_i_|Z_i_* = 1] *− E*[*D_i_|Z_i_* = 0] ≠ 0.

Under these assumptions, we can define an instrumental variable parameter as
10
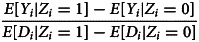
 which, if the treatment effect does not vary across units, is equivalent to the two-stage least square parameter described in the main text
11
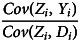

Note, the numerator of Equation (10) (i.e. the effect of the instrument on the outcome, *E*[*Y_i_|Z_i_* = 1]*−E*[*Y_i_|Z_i_* = 0]) is often referred to as ‘reduced form’ or ‘intention to treat’, and it requires only the unconfoundedness of *Z_i_ vis-à-vis* the outcome *Y_i_* to be interpreted causally.

If the treatment effect varies across units and an assumption known as *monotonicity* holds (i.e. no unit does the opposite of its instrument assignment: *D_i_*(1) ≥ *D_i_*(0), ∀*i*), then Equation (10) can be interpreted as
12
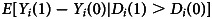

Equation (12) defines a local average treatment effect, that is, the effect of the cultural trait on the units that responded to the instrument *Z_i_*. This is an important – although not necessarily positive – result, which implies that instrumental variable estimation (under the four assumptions) identifies an average treatment effect only on a specific subpopulation of units (Angrist et al., 1996).

To answer this question, Gorodnichenko and Roland (2017) rely on instrumental variable estimation. Among the various instruments used by the authors, for the sake of brevity, here we focus only on two of them: historical pathogen prevalence (Murray & Schaller, 2010) and a genetic marker based on Cavalli-Sforza et al.'s (1994) data measuring the Mahalanobis distance between the frequency of blood types in a given country and the frequency of blood types in the UK (one the most individualistic country in their sample). The rationale behind the pathogen prevalence instrument echoes a functional view of culture (cf. Nettle et al., 2013), which suggests that collectivism might be a cultural adaptation to a pathogen-ridden ecology. Where pathogens abound, norms of out-group discrimination, limitations to individual behaviours and internalisation of group interests could favour individual-level survival chances. Different from the pathogen prevalence measure, the genetic instrument hinges on a purely cultural transmission argument. The intuition is as follows. Parents transmit their blood type to their children, but they also spread their culture, individualism included. As such, blood type and culture (or, at least, the vertically culturally transmitted portion of it) should correlate. That is, the argument is not that blood types cause culture, but rather that distance between the frequency of blood types across countries can serve as a proxy for differences in cultural traits across countries.

Overall, the results of Gorodnichenko and Roland (2017) suggest that individualism causes economic development, as measured by the logarithm of income per worker. However, can we trust these results? Both instruments predict Hofstede's individualism–collectivism index well, so they both meet the relevance condition. Concerning the independence assumption, more circumspection is required, as neither instrument is directly assigned at random by the researcher. As-if randomness might be plausible for the genetic instrument, to the extent that it is hard to imagine a specific omitted common cause that could jointly cause blood type, individualism and economic development. Yet independence is perhaps less clearly met for pathogen prevalence, as unobserved geo-climatic variables might act as confounders (e.g. humidity, distance from the equator, temperature). It is, thus, reassuring that the instrumental variable results of Gorodnichenko and Roland (2017) survive the inclusion of absolute latitude and longitude, thus providing some tentative evidence for (conditional) independence. Let us now consider exclusion. While a direct effect of the genetic-based instrument and of pathogens on current economic outcomes might be far-fetched, exclusion could also be violated if, for instance, either instrument causes economic development via some causal channels different from individualism–collectivism, like institutional quality or some alternative cultural traits (see, e.g. Acemoglu et al., 2001; Nash & Patel, 2019). Again, the authors control for several suspicious covariates, thus providing some confidence that the main results are not explained away by alternative causal channels like the percentage of the population practising different religions or institutional quality.

Finally, and irrespective of the validity of the instruments, it should be noted that the results of Gorodnichenko and Roland (2017) do not directly address issues related to the cultural relatedness of different countries (see Mace et al., 1994). The issue is that different countries should perhaps not be considered and analysed as independent data points, given their common cultural origin (e.g. the UK and Australia). Phylogenetic methods are probably the only clear-cut solution to the issue, even though Gorodnichenko and Roland (2017) conduct a series of robustness tests that also speak to this issue. Specifically, the authors show that their baseline results are confirmed also when restricting attention to countries with historically high shares of indigenous populations. This pattern suggests that the individualism–economic development relationship is not solely driven by European migration patterns that could have brought individualistic values to the US, Australia and other parts of the world. The instrumental variable estimation results also hold within continents, suggesting that the individualism–wealth relationship is not only driven by some macro geo-cultural area.

*Geography, history and their interactions*. Finding valid instruments is often more of an art than a science, requiring substantial subject-matter expertise and creativity. Indeed, a good instrument is often a variable ‘that you would never think to include in a model of the outcome variable, and in fact you may be surprised to find that it ever had anything to do with assigning treatment’ (Huntington-Klein, 2021, chapter 19). It is, thus, impossible to formulate some specific guidelines on where to find instruments, yet some ideas related to instrumental variables and culture (and cultural persistence, specifically) can be found in several articles that we briefly touch upon.

For instance, some authors have relied on instruments based on geo-climatic variables to study how traditional subsistence systems have led to the emergence of norms related to individualism and collectivism, as well as obedience, leadership, and gender roles (e.g. Alesina et al., 2013; Buggle, 2020; Lonati, 2020; Talhelm et al., 2014). The intuition here is that specific environmental conditions predict whether entire societies develop a specific subsistence system. These systems, in turn, are linked to the emergence of specific cultural traits, which tend to be then transmitted over time (e.g. herding favouring autonomy and agriculture favouring interdependence; see Nisbett et al., 2001; Uskul et al., 2008). Other authors have used geographic instruments to study the persistent effect of traumatic historical events. For instance, Nunn and Wantchekon (2011) study the negative effects of the slave trade in Africa on current norms of trust by instrumenting the historical slave export data by distance from the coast of different locations in the continent (i.e. the more distant from the coast a location is, the lower the chance it experienced the slave trade; see also Pierce & Snyder, 2017; Teso, 2019).

Note that these examples cannot pinpoint a clear-cut cultural story thanks to instrumental variables alone, as the long-term effects of subsistence systems (as instrumented by geo-climatic conditions) or of the slave trade (as instrumented by distance from the coast) could also be sustained by non-cultural causes, like institutions, economic systems and demography. Yet instrumental variable estimation can provide some suggestive evidence related to culture and cultural transmission. In turn, we hope that these examples can offer some clever ideas on how researchers can find plausibly valid instruments.

#### 3.1.4. More advanced considerations

Instrumental variable estimation allows the identification of the causal effect of culture even if the culture–outcome relationship of interest is confounded. This result is remarkable but comes at a hefty price: to relax the unconfoundedness assumption, researchers need to invoke three new assumptions, some of which are untestable and by no means weaker than unconfoundedness. This does not seem like a good bargain, so why would researchers ever want to use instruments?

First, we invite our readers to see instrumental variable estimation not as a substitute – but rather as a complement – to strategies based on unconfoundedness and positivity. As these two approaches rely on different assumptions, finding converging evidence for a causal effect of culture with both of them might indicate a particularly credible result. Second, while independence and exclusion are ultimately untestable, some statistical tests (Wooldridge, 2002, chapter 15), sensitivity analyses (Conley et al., 2012) and *ad-hoc* falsification tests (see e.g. Nunn & Wantchekon, 2011) can be used to bolster the credibility of an instrument. Third, even when the exclusion assumption is not satisfied, the effect of the instrument on the outcome can still have a causal meaning – the so-called ‘intention to treat’ effect. For instance, even if the instrument affects the outcome directly (Figure 3d), the intention to treat is unbiased, effectively representing the effect of being encouraged to take the treatment rather than the effect of treatment intake *per se*. Note, however, that the intention to treat still relies on the (conditional) independence of the instrument. That is, if the instrument is not as good as random (as in Figure 3c and f), the intention to treat will not be causally interpretable.

### 3.2. From randomised experiments to natural experiments: Spatial regression discontinuity design and related approaches

A major difficulty when studying culture is that groups that possess different cultural traits usually live in different ecological, demographic, institutional and economic environments. Intuitively, however, the more similar the environment where different cultural groups live, the lower the chance of confounding (see Cohen, 2019; Uskul et al., 2008). In this section, we review the spatial regression discontinuity design and related approaches, that is, designs that push this intuition to its extreme by comparing units that live in the neighbourhood of a border that separates cultural groups sharply.

#### 3.2.1. Identification assumptions

In general terms, regression discontinuity design leverages variation generated by a cutoff that sharply separates two groups, one treated and one untreated (see the pioneering work of Thistlethwaite & Campbell, 1960). Regression discontinuity design applications usually rely on discontinuities in time (e.g. before vs. after an unexpected terroristic attack; see Bastardoz et al., 2022), thresholds set by law or public authorities (e.g. Medicare eligibility at age 65, see Card et al., 2008), or vote shares necessary to obtain a majority (e.g. Flammer, 2015; Lee, 2008). When studying culture as a cause, however, the appeal of the regression discontinuity design becomes clearest in spatial terms, wherein a specific geographical border might assign different units to different cultural groups while holding constant most other conditions, thus representing a natural experiment that approximates a physical randomisation of culture.

**Figure 4.**
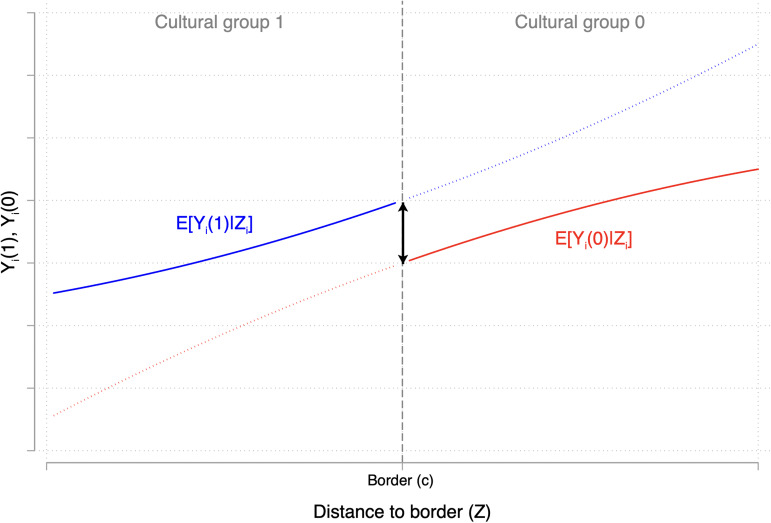
Regression discontinuity: a representation (see Cattaneo et al., 2023). *Note:* The solid curves represent observed outcomes; the dotted curves represent unobserved outcomes.

More formally, a spatial regression discontinuity design is a design where all units have a score (often labelled ‘running’ or ‘forcing’ variable) representing their distance from a border. This border is known *ex-ante* to separate two cultural groups, thus the process of assigning units to either group is completely known. If this score is above the cutoff, a unit is assigned to one cultural group. If the score is below the cutoff, the unit is assigned to another cultural group. Thus, the jump exhibited by the observed outcomes of the two groups exactly at the cutoff can be used to estimate the difference in the groups’ potential outcomes (Cattaneo et al., 2023; Imbens & Lemieux, 2008). Graphically, this local average treatment effect is represented by the local jump in the two solid curves of Figure 4.

**Figure 5.**
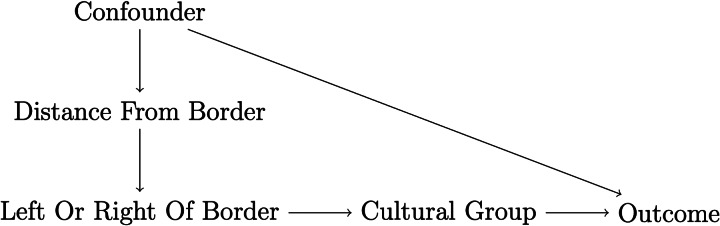
Regression discontinuity: an alternative representation (see Huntington-Klein, 2021).

In a spatial regression discontinuity design, the key identification assumption is that cultural affiliation to a group (i.e. the treatment) is the only factor that varies discontinuously at the cutoff (see Technical box 4). This assumption is conceptually similar to unconfoundedness because it requires all units’ characteristics (both observed and unobserved) other than culture to be similar in the vicinity of the cutoff. This so-called ‘continuity assumption’ (Hahn et al., 2001) can be visualised in Figure 4, wherein the same-coloured curves do not jump discontinuously at the cutoff. Another way to think about continuity is in the graph of Figure 5 (Huntington-Klein, 2021): regression discontinuity design does not assume that the score is as good as random, but rather that being on the left or the right side of the cultural border is (see also Cunningham, 2021; Steiner et al., 2017).

Continuity is not a weak assumption and is ultimately untestable. When studying culture as cause, continuity would intuitively be met if all units were effectively randomised to live on either side of the border. Yet individuals are probably able – and willing – to sort on their preferred side of the border (see e.g. Aepli et al., 2021). Yet isn't this a major violation of spatial regression discontinuity design assumptions? Quite probably it is. However, the regression discontinuity intuition can be still useful, even without a formal spatial regression discontinuity design and without its formal properties (Keele & Titiunik, 2016). Specifically, limiting the analysis to adjacent units might not allow completely clean causal estimates, but it will usually mitigate major confounding issues by design – a so-called ‘conditional local geographic ignorability’ design (e.g. Aepli et al., 2021; Eugster et al., 2017; Keele & Titiunik, 2016). This strategy can be thought of as a form of design-based approach (Card, 2022; Keele, 2015) that mixes the spatial regression discontinuity design intuition with the selection on observables one.

#### 3.2.2. Estimation: Basic notions

Parametric estimation is the most intuitive way to understand the logic of spatial regression discontinuity design and related approaches. In a nutshell, regression discontinuity design requires either fitting two regression lines – one on the left and one on the right of the cutoff – or one regression line with an interaction term of the form
13
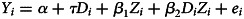
where *Y_i_* is the outcome of interest, *Z_i_* is the distance from the border, *D_i_* is an indicator of the cultural group membership and *e_i_* is an unobserved disturbance (i.e. all unmodelled factors affecting *Y_i_*). The main parameter of interest is *τ* (i.e. the effect of being in one or the other cultural group), while *β*_1_ measures if the distance from the border associates with the outcome on one side of the border, and the parameter *β*_2_ allows for a different effect of distance from the border on the other side of the border. Of course, more complex versions of this model are also possible (e.g. powers of *Z_i_*, see, e.g. Angrist & Pischke, 2014).

As the identifying assumption of spatial regression discontinuity design (as well as the logic of the conditional local geographic ignorability design) can be valid only in the vicinity of the threshold, this regression should only be run on observations that lie in the vicinity of the cultural border and not over the entire domain of the score. To show the robustness of their results, researchers usually run the regression in Equation (13) using various windows of data, test the robustness of their regression specification using polynomials for *D_i_* (but see Gelman & Imbens, 2019) and/or use a specific non-parametric technique that puts more weight on observations closer to the cutoff (i.e. kernel regression). Concerning statistical inference, calculations of standard errors in spatial regression discontinuity design are a rather delicate matter; we thus re-direct readers interested in this topic to Cattaneo and Titiunik (2022) or Cattaneo et al. (2020).

Technical box 4: Regression discontinuity design

Let *Z_i_* be the running variable. Units are assigned either to the cultural group *D_i_* = 1 if *Z_i_* ≥ *c* or to *D_i_* = 0 otherwise, where *c* denotes the cutoff point/border. In this scenario, unconfoundedness holds trivially, because *D_i_* is a deterministic function of the running variable *Z_i_*. However, positivity never holds, because *P* [*D_i_* = 1*|Z_i_* = *z*] is either 0 or 1 (Imbens & Lemieux, 2008). Identification, thus, relies on a different assumption, known as continuity (Hahn et al., 2001):
14
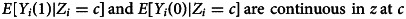

Formally, the continuity assumption allows us to define the estimand of interest as,
15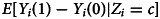 which is identified by
16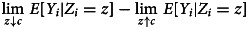 where ↓ (resp. ↑) means that *z* is approaching *c* from above (resp. from below). Equations (15) and (16) describe a local causal effect in that they focus on the difference between the potential outcomes *precisely* at the cutoff *c*. Thus, much like the parameter identified by instrumental variable estimation, regression discontinuity design identifies a local average treatment effect.

For completeness, note that a different school of thought suggests seeing regression discontinuity design as a local experiment, thus implying a more demanding assumption of as-if randomisation in a small window around the cutoff. We do not cover these differences in this paper, re-directing interested readers to De la Cuesta and Imai (2016) and Cattaneo and Titiunik (2022).

Finally, note the difference between a standard regression discontinuity design and both its geographic (or spatial) version and the conditional local geographic ignorability design. In a geographic regression discontinuity design, the location of each unit *i* is determined by two coordinates (e.g. latitude and longitude), which define a set of cutoff points along a border. In a conditional local geographic ignorability design, the continuity assumption is not directly invoked, but the vicinity to the cutoff is used as a way to approximate (conditional) unconfnoundedness by design, in that it is more reasonable to assume the independence of the potential outcomes for units closer to the cutoff rather than for units distant from it.

#### 3.2.3. Where to find cultural discontinuities?

Different cultural groups are often divided more or less sharply by administrative borders (e.g. country or state borders). Using these cutoffs for a spatial regression discontinuity design might seem tempting, but care is required. The problem is that groups separated by these borders are not only culturally different but also experience different laws, economic conditions, histories, etc. This leads to confusion: is this geographical contrast identifying the effect of culture, some institutional difference, or other differences? For this reason, we believe that the most convincing types of cultural discontinuities are often found *within* the same administrative unit. We discuss some such examples below.

*The Röstigraben in Switzerland.* A cultural cutoff that is sometimes discussed in the economics literature is the linguistic border in Switzerland (e.g. Cottier, 2018; Eugster et al., 2011; Gentili et al., 2017). Switzerland is a small European country divided into several autonomous administrative regions, so-called ‘cantons’. Most cantons belong to one of the country's two main linguistic/cultural groups: Romance (i.e. French and Italian, living for instance in Lausanne or Lugano) or German (e.g. Zurich). Despite their small size and geographical proximity, these cantons sometimes exhibit strikingly different attitudes and values reflected in important aggregated outcomes, such as voting patterns and labour-market conditions. A natural question is whether culture causes these differences. However, directly comparing Romance- and German-speaking cantons is not a particularly convincing empirical strategy. The problem is that, while cantons share major infrastructures, several federal laws and generally prosperous economic conditions, they also vary importantly in geographic and demographic factors, gross domestic product per capita, local constitutions, policing and judiciary systems. So, how to identify the causal effect of culture in this case?

Luckily, a handful of cantons are divided down the middle by a centuries-old cultural border, the so-called *Röstigraben*. The *Rösti* is a potato-based dish typical of the German-speaking cantons, and a literal translation of *der Röstigraben* would be something like ‘the hash brown ditch’. This linguistic/cultural border represents a distinctive empirical opportunity. Living in the same administrative unit, individuals share similar ecological, demographic, economic and institutional environments, yet they are embedded in two different linguistic/cultural groups. Intuitively, the similarity between these culturally different individuals will be maximal around the *Röstigraben*.

Eugster et al. (2017) leverage exactly this intuition, pursuing a conditional local geographic ignorability design to study the cultural causes of unemployment duration in these two groups. After having lost a job, Romance speakers tend to stay unemployed for longer than German speakers, hinting at systematically different attitudes towards work. To isolate the role of these socially transmitted norms and beliefs, the authors compare job seekers only in the vicinity of the *Röstigraben*, restricting their attention to individuals living up to 50 km away (in terms of road distance) from the cultural border. Their results highlight a sizeable and robust effect of the linguistic border, consistent with an effect of culture net of environmental confounds.

Is this result causally interpretable? The *Röstigraben* contrast would be shaky if individuals living on either side of the cultural border were systematically different in some non-cultural factors. For instance, if German speakers were more educated than the Romance ones, they could more easily obtain jobs because of non-cultural factors. Similarly, if German speakers were to live in a richer economy, they might have more job offers owing, again, to non-cultural factors. Such scenarios are not implausible, as the descriptive evidence of Eugster et al. (2017) suggests that some individual and municipality characteristics are unbalanced in the two cultural groups, even when limiting the attention only to observations in the vicinity of the *Röstigraben*. To reduce such concerns, Eugster et al. (2017) include several covariates measuring potentially problematic individual characteristics (e.g. individual qualifications) and municipalities’ characteristics (e.g. demographic structure, median wage), as well as other potential confounds (e.g. year of interview, city of residence dummies). As a result, this study might not decisively conclude that cultural traits related to job attitudes are the only factor that drives the different behaviours of German- and Romance-speaking Swiss, but it can at least show that the explanatory power of alternative non-cultural explanations (e.g. economic conditions) seems too small to be the sole reason for the observed discontinuity between the two groups.

Finally, from a cultural transmission viewpoint, three points are noteworthy. First, the already mentioned issue of cultural non-independence (Mace et al., 1994) is almost certainly present in this setting. However, we believe that it is unlikely to drive the results reported by Eugster et al. (2017). The non-independence argument suggests that Romance- and German-speaking Swiss should be culturally similar, as they probably share extensive cultural ancestry. Yet the fact that one finds a difference rather than a similarity between these groups suggests that culture plays a role here *despite* the cultural non-independence. Second, Eugster et al. (2017) cannot pinpoint the exact cultural trait responsible for the difference. Rather, by holding the environment constant, the article infers that differences in unemployment between the Romance and German groups must reflect a cultural trait related to attitudes to work. To this extent, spatial regression discontinuity design and similar approaches are especially appropriate if researchers aim at identifying a broad constellation of cultural traits responsible for a given effect, but might be insufficient if researchers are interested in a very specific cultural trait. Last, spatial regression discontinuity design and similar approaches usually do not allow differentiation between vertical and horizontal (or oblique) cultural transmission. As culture changes sharply at the border for all individuals embedded in a group, researchers cannot identify the effect of, say, coming from a German-speaking family (i.e. vertical transmission) net of the effect of living among German-speakers (i.e. horizontal and oblique transmission).

*Discontinuities based on historical borders.* Finding credible cultural discontinuities is no easy task and requires subject-matter expertise, as well as data availability. Aside from relying on within-country linguistic or ethnic borders similar to the *Röstigraben* (see Moscona et al., 2020), researchers might find it useful to look for borders that existed in the past, like historical administrative boundaries (Becker et al., 2016; Lowes et al., 2017; Testa, 2021). These historical borders separated areas that experienced different institutions, economic conditions and cultural traits in the past, but are now part of the same country or district and, thus, share a similar socio-economic environment. As such, finding evidence of behavioural differences in groups separated by historical administrative borders suggests that historical cultural differences might have persisted till this date.

#### 3.2.4. More advanced considerations

Using geo-cultural cutoffs as an identification strategy is an intuitively compelling and conceptually straightforward way to make causal claims about culture. However, special care is needed when using geographical borders as discontinuities. These difficulties are potentially serious, so we briefly cover them here, re-directing our readers to Keele and Titiunik (2015, 2016) and Keele et al. (2015) for more details.

The first issue relates to the very definition of ‘distance from a border’, which we discussed in our paper as an absolute scalar measure, but should be thought of as a multidimensional one (i.e. longitude and latitude) if one wants to strictly apply a spatial regression discontinuity design. The second issue relates to measurement error in the distance from border measure (i.e. it is often hard to locate precisely a unit) and to the possibility of observations clustering around the cutoff (i.e. most units might live in a specific location, like a city or a village). The third issue concerns the fact that geo-cultural borders are rarely – if ever – deterministic cutoffs. Rather, they can be often thought of as ‘nudges’ that increase the probability of an individual belonging to a given cultural group. If that is the case, the so-called ‘fuzzy regression discontinuity design’ can be more appropriate (i.e. a technique wherein the cutoff does not determine cultural affiliation directly, but increases the probability of belonging to a cultural group).

The last, and probably most important, difficulty revolves around the key assumption of spatial regression discontinuity design and related strategies, which requires units around the cultural border to be ‘virtual clones’, similar to a randomised experiment. This assumption is ultimately untestable and can be a heroic one to make. Subject-matter expertise is, thus, required to make this call. Still, there are several empirical ways to probe its plausibility. For instance, researchers can compare the distribution of observable covariates for units on either side of the border, hoping to see a balanced distribution around the cutoff (see e.g. Abadie & Cattaneo, 2018). A similar falsification test revolves around showing in a plot if suspicious covariates jump around the threshold. If some jumps are detected, or if some imbalances are found in the vicinity of the border, this signals that the continuity assumption might be violated and that individuals might actively manipulate their exact location around the cutoff (cf. Imbens & Lemieux, 2008). Researchers can also test whether placebo cutoffs (which should assign units to no specific treatment) actually have no effect, thus bolstering the idea that treatment is determined only by the distance from the threshold chosen (for more details, see Cattaneo & Titiunik, 2022; Cattaneo et al., 2020).

### 3.3. From randomised experiments to common garden experiments: Epidemiological approach

In this section, we turn our attention to a family of research designs that are sometimes called the ‘epidemiological approach’ in economics (Fernández, 2011). In general terms, the epidemiological approach refers to any research design that tries ‘to identify the effect of culture through the variation in [an outcome] … of individuals who share the same economic and institutional environment, but whose social beliefs are potentially different’ (p. 489, Fernández, 2011). This intuition is similar to the regression discontinuity design one, where researchers compare units with different cultural affiliations living in similar environments. Yet the epidemiological approach does not focus on units that are geographically segregated, but rather on culturally heterogeneous individuals that live in the same location – immigrants or their native-born descendants.

Note, the epidemiological approach is not a canonical identification strategy. That is, different from instrumental variable estimation and regression discontinuity design, the epidemiological approach is just another empirical strategy that relies on unconfoundedness and positivity. For cultural evolutionary scholars, however, this design represents a distinctive opportunity, allowing under some assumptions to separate the effect of the environment where a cultural trait emerged from the effect of culture proper.

#### 3.3.1. Identification assumptions

The epidemiological approach intuition echoes a design sometimes used by epidemiologists (hence the name), who try to separate the genetic and environmental contributions of some medical conditions by contrasting immigrants to natives. When applied to culture, the logic is similar, although researchers focus on the cultural (rather than genetic) contribution net of the environment where individuals live. Thus, the notional experiment the epidemiological approach tries to approximate is one in which individuals from culturally diverse groups were to be moved randomly to a single common environment, so as to observe their behaviours in the same environment. If immigrants’ behaviours remain identical to the ones they exhibit in their native environment, then the culture is a plausible cause of behaviours. If, on the contrary, immigrants from different cultural groups behave identically in the new environment, then the environment is the plausible cause of behaviours.

Here lie both the strengths and the weaknesses of the epidemiological approach. Compared to a strategy based on purely observational data and statistical adjustment, the epidemiological approach can minimise environmental confounding by design. Yet violations of unconfoundedness do not only arise from environmental factors but can emerge because of other omitted common causes (e.g. characteristics of the individuals) or because of selection problems. Both issues stem from the fact that cultural evolutionary researchers clearly cannot run a ‘common garden’ or ‘transplant’ experiment with individuals (cf. Atran et al., 2002). Rather, they merely observe the behaviours of individuals who have decided, at least partly, whether they want to move out from their original environment and where they prefer to go. Given this inherently observational nature, any use of the epidemiological approach should be very well thought through and researchers should examine if and why they feel confounding is not a major issue in the context at hand.

#### 3.3.2. Estimation: Basic notions

Estimation of epidemiological approach-type strategies follows essentially the same principles of any strategy based on unconfoundedness and positivity. Specifically, the epidemiological approach is usually estimated via a regression of the form:
17
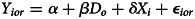
where *Y_ior_* is an outcome of interest for an individual *i* having an environment of origin *o* and an environment of residence *r* (where *o* ≠ *r*), *D_o_* is a cultural trait of interest present in the environment of origin, *X_i_* are some covariates used to meet the unconfoundedness assumption and *ɛ_ior_* is the disturbance (i.e. all unmodelled factors affecting *Y_ior_*). *β* is the parameter measuring if culture affects individuals’ choices in the novel environment of residence.

#### 3.3.3. Where to find different cultural groups in the same environment?

**Figure 6.**
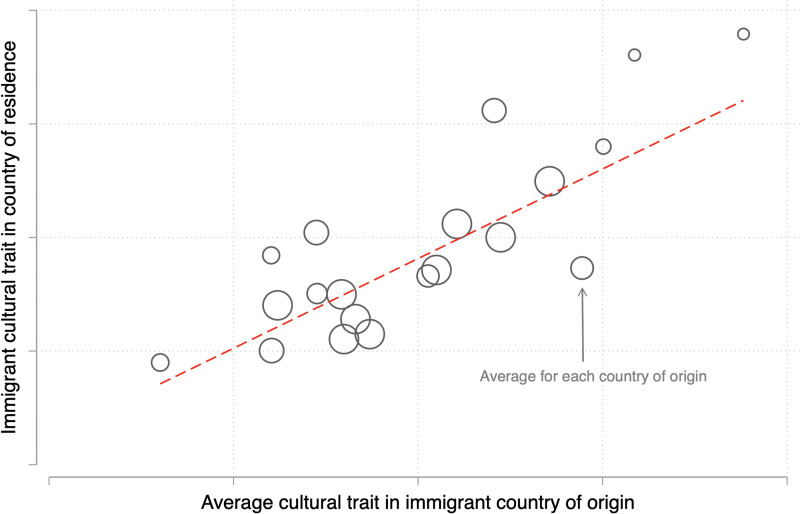
Epidemiological approach using immigrants’ data: a representation (see Luttmer & Singhal, 2011). *Note:* Each circle represents the average observations of immigrants coming from a given country. The dimensions of circles represent a different number of migrants observed.

*Immigrants and immigrants’ descendants.* A popular way to implement the epidemiological approach is with immigrants’ data (for an intuitive graphical representation, see Figure 6). However, many researchers do not focus only on first generations (i.e. individuals who live in a given country or region, yet are born in a different one), but rather study second generations, that is, native-born children of immigrants. There are several reasons for this choice. For starters, second-generation immigrants are likely to have fewer direct connections with their country of origin compared to first generations, thus making the culture of origin–environment of residence separation more clear-cut. Moreover, it is often unrealistic to assume that first-generation immigrants are as-if randomised to a destination country. Rather, it seems plausible that these immigrants might actively choose their country of residence based on geographical or cultural proximity with their country of origin, economic development in the country of destination, and other factors (Fernández, 2011). Such concerns are less obviously relevant for second generations, who do not directly decide where to live or whether or not to carry the culture of origin of their parents.

Immigrants data in an epidemiological approach-type framework have been used in economics to study predictors and outcomes as diverse as family ties (Alesina & Giuliano, 2010), preference for redistribution (Luttmer & Singhal, 2011), kinship structures (Enke, 2019), traditionalism (Giuliano & Nunn, 2021), attitudes related to gender roles (Alesina et al., 2013) and group conformity (Buggle, 2020). Cultural evolutionary scholars have also sometimes employed variants of the epidemiological approach to study psychological processes (Mesoudi et al., 2016) and attitudes towards female genital cutting (Vogt et al., 2017). In a particularly impactful piece that we now discuss more in detail, Fernández and Fogli (2009) use census data to study whether the fertility rate and worked hours of 30–40 US-born married women having foreign-born parents are explained by the fertility rate and female labour force participation in their parents’ countries of origin. The authors find robust relationships between these variables, suggesting that culture causes these patterns.

Three potential issues could in principle hamper the causal interpretability of Fernández and Fogli's (2009) results. First, immigrants from different cultural groups might be systematically different for reasons other than culture (e.g. demographic characteristics, education levels). To deal with this concern, Fernández and Fogli include several individual-level covariates that might represent potential common causes, like women's education level, parental education and age. Note that these covariates might also be ‘bad controls’, to the extent that they might be caused by the culture of origin (cf. Cinelli et al., 2022). Thus, including them might be akin to adjusting for a mediator (something we should usually avoid doing, see e.g. Major-Smith, 2023). At worst, adjusting for these covariates might even lead to collider bias owing to conditioning on common effects. It is thus reassuring to see that Fernández and Fogli's (2009) results emerge both when adjusting or not adjusting for these variables.

Second, the environments where immigrants currently live might still be not so comparable. For instance, even if two immigrants live in the same country of residence, they might still be exposed to completely different contexts (e.g. large city vs. rural area). To address this potential concern, Fernández and Fogli (2009) adjust in a regression framework for metropolitan standard areas’ fixed effects where the sampled women live, effectively comparing women in the same area. Relatedly, immigrants might re-create some of the institutional conditions typical of their home country in their new country of residence. This might be especially the case if migrants’ location choices are predicted by previous settlement patterns, causing immigrants to reside in geographical clusters (e.g. Little Italy, Chinatown). Indeed, Fernández and Fogli (2009) find that the effect of fertility in the father's country of origin is stronger if a woman comes from a cultural group that tends to cluster in a given area, suggesting that either a horizontal cultural transmission or an institutional pressure is at play. Note, however, that ethnic density is not solely responsible for the results.

Last, immigrants might be a non-random sample of the inhabitants of their country of origin (Borjas et al., 1992). For instance, immigrants might be more or less skilled, rich or educated than their non-migrated compatriots. This issue certainly hampers the generalisability of the findings, in that Fernández and Fogli (2009) are not studying the effect of culture in the general population, but rather in a specific sub-group composed only of immigrants. Yet this issue might also threaten causal identification if the studied cultural trait affects the decision to migrate (and, thus, the probability of being observed by the researchers), leading to potential issues related to conditioning on a common effect.

From a cultural evolutionary viewpoint, two additional points are worth mentioning. First, an epidemiological approach strategy like the one used by Fernández and Fogli (2009) is bound to find evidence that culture does *not* matter. As the cultural trait of interest is assumed to be transmitted only from parents (i.e. vertical cultural transmission), finding no effect of culture of origin does not mean that culture does not matter, but rather that vertically transmitted culture does not play a major role. Conversely, finding evidence that culture of origin matters does not mean that horizontal and oblique transmission channels are not at play. Second, using an epidemiological approach strategy based on immigrants coming from different countries of origin/residence implicitly assumes that different countries are separate units of analysis. This might lead to some conceptual issues (i.e. country and culture are not synonyms), as well as to the already-mentioned issues related to common cultural ancestry (Mace et al., 1994). Note, however, that the epidemiological approach offers some ways to circumvent this issue, by explicitly considering the geographical, historical and cultural distance between a migrant's country of origin and the country of destination.

*Epidemiological approach beyond immigrants’ data*. Employing immigrant data is a particularly intuitive way to apply the epidemiological approach. Yet it is not the only one. Some applications are particularly creative. For instance, Fisman and Miguel (2007) study whether culture causes corruption by examining variations in United Nations diplomats’ parking violations. The identification strategy is as follows. United Nations diplomats belong to different cultural groups, yet they all work in the same environment (i.e. Manhattan) and, crucially, benefit from diplomatic immunity. This means that any difference in their misbehaviour – which the authors clearly document – cannot be due to environmental differences or fear of punishment (i.e. institutions, cf. Powers et al., 2016), but could be caused by culture.

A more recent application of an epidemiological approach-type strategy is Frake and Harmon (2023). In their paper, the authors study the emergence and further transmission of a culture of misconduct at the Chicago Police Department. Specifically, the paper studies whether young police recruits – who must undergo a long and intense academy training – engage in more misconducts (e.g. illegal searches, falsification of evidence) if they are exposed during their training to misconduct-prone peers and if, once promoted to managers, they transmit their misconduct culture to their subordinates. To eliminate several issues related to selection and omitted common causes, the authors leverage the fact that young recruits are randomly assigned (i.e. a lottery) to a training cohort and, thus, to a culture of misconduct to begin with. Their results highlight both a horizontal transmission of misconduct and an inter-generational one.

#### 3.3.4. More advanced considerations

The design features of the epidemiological approach make it a relatively credible identification strategy in the context of studying culture as cause. However, it is important to reiterate that the epidemiological approach still faces all the potential limitations typical of strategies relying on unconfoundedness and positivity. Solving these issues by mixing the epidemiological approach logic with quasi-experiments is in principle possible (e.g. exogenous shocks in migrants’ locations owing to country of origin or country of destination factors), but such exogenous sources of variation are certainly hard to find (cf. Frake & Harmon, 2023). We, thus, invite cultural evolutionary scholars to use the epidemiological approach while minding its potential weaknesses.

## 4. Important topics we did not cover

In this paper, we have discussed common challenges and opportunities related to the study of culture as cause. The details behind the various designs or techniques we reviewed are widely different, yet the overall message we hope to have conveyed is simple. Causal inference is chiefly a conceptual matter, which requires more theoretical clarity, transparency about the assumptions one is ready to make and a firm grasp of the data at hand rather than technical sophistication or statistics *per se*. Following this blueprint, in our paper we glossed over several statistical ‘details’, preferring to focus on the big picture of causal identification and on practical examples. As a result, our review is necessarily cursory and is by no means a complete or detailed survey of techniques and notions related to causal inference. There are, however, some key issues, topics, and ideas that space limitations prevented us from covering in detail, but are briefly discussed in this section.

### 4.1. No-interference

A topic we only briefly touched upon is the no-interference assumption. Much like consistency, no-interference can be easily overlooked, yet represents a conceptual and empirical concern that should be considered carefully when studying culture as cause.

Whether no-interference violations represent a major problem depends, however, on the setting at hand. If researchers study micro-evolutionary processes (i.e. how single individuals transmit cultural traits to each other), violations of no-interference are expected almost by definition (An & VanderWeele, 2022; VanderWeele & An, 2013). For instance, unwanted treatment spillovers might emerge if individuals who learned a bit of socially transmitted information (i.e. the treatment group) interact with individuals without that information (i.e. the control group), possibly influencing their outcome (cf. Vogt et al., 2016). However, if one focuses on more macro levels of analysis (e.g. whether some group-typical, learned information affects group-typical outcomes), no-interference issues might be less salient. For instance, if researchers study the effect of individualism on economic development in a cross-section of countries, it might be fair to assume that the individualism level of, say, Germany will not importantly affect the economic outcomes of, say, Indonesia. Similarly, if one studies the effect of culture around the *Röstigraben*, violations of no-interference are easy to imagine (e.g. the German cultural affiliation status of a village might affect the economic outcome of the neighbouring Romance villages, too). Yet these violations are likely to depress the chance of finding a true effect of culture rather than increasing the chance of finding a spurious one, thus reinforcing the cultural interpretation of some of the results we reviewed (e.g. Eugster et al., 2017).

### 4.2. Other canonical research designs

In this paper, we reviewed only a selective set of research designs relevant to causal identification. Yet other canonical designs could also be useful for cultural evolutionary scholars. For instance, we did not discuss fixed effects estimation (see Bell & Jones, 2015; Imai & Kim, 2021), difference-in-differences studies (for a recent review, see Roth et al., 2022) and the synthetic control method (Abadie et al., 2011, 2015). These strategies rely on different types of selection on observables identification assumptions and can be used when the data collected by researchers not only contain a cross-sectional component but also a time-based or hierarchical dimension (e.g. individuals nested in groups, units observed over time).

### 4.3. Other identification threats

This paper covered the main identification threats that might emerge when studying culture as cause. However, we certainly did not cover all possible sources of bias.

For instance, we have not reviewed the issue of measurement error in the treatment variable. Measurement error (i.e. differences between an observed variable and its true, latent value) can cause bias (Wooldridge, 2002) and is a topic that can be particularly relevant for cultural evolutionary researchers, who might often need to analyse ill-measured predictors. Another identification threat we have not touched upon is missing data (Enders, 2022; Little & Rubin, 2019). While this issue shares some similarities with selection bias (see Elwert & Winship, 2014), it is a conceptually different difficulty that might emerge when analysing data with missing responses in a questionnaire or missing ethnographic observation for some societies. If missingness is not a random event and is treated incorrectly (e.g. listwise deletion of observations), it will cause bias. Moreover, we have not discussed the many difficulties related to causal mediation analysis, an empirical strategy that can in principle allow researchers to identify the mechanism through which an estimated effect of culture comes about (for details, see Imai et al., 2010). Finally, when introducing the randomised control trial as the ideal design to make causal claims, we have purposefully abstracted from common identification threats that might emerge when running experiments, like imperfect compliance to treatment (see, e.g. Sagarin et al., 2014), unbalanced representations of confounders owing to small samples (Deaton & Cartwright, 2018), non-random attrition (Duflo et al., 2007) or demand effects (Zizzo, 2010). These and other issues make it clear that causal identification is not trivial in practice even when relying on ideal designs.

### 4.4. Statistical estimation and inferential mode

In this paper, we did not discuss details about the actual estimation of causal effects (e.g. regression, matching, inverse probability weighting; Keele et al., 2020). Similarly, our paper has not covered different modes of inference, like Bayesian causal inference (for a review of Bayesian causal inference rooted in the potential outcome framework, see, e.g. Li et al., 2023). Moreover, we have not touched upon any technicalities (e.g. inferential tests, tests of model fit) or practicalities (e.g. software implementation, see, e.g. Cunningham, 2021). Yet these ‘details’ are clearly important. For instance, the way in which standard errors or other measures of statistical uncertainty are calculated can make all the difference when observations are not independent of each other (e.g. repeated observations for the same units, individuals nested in groups; see e.g. Cameron & Miller, 2015; McNeish & Kelley, 2019). This issue is also related to the cultural non-independence problem (Currie, 2013; Mace & Holden, 2005).

### 4.5. Potential outcomes and beyond

Finally, while our paper used the potential outcome framework as its backbone, we should stress that other logical–statistical tools can also be used to guide researchers’ causal reasoning, like DAGs (Pearl, 2010b) or classic population regression models and exogeneity conditions found in econometrics textbooks (Wooldridge, 2002). While *prima facie* very different, these frameworks are conceptually similar and are largely complementary. For instance, DAGs are particularly useful to motivate covariate selection, whereas the potential outcome framework is not so helpful in this regard. On the flip side, DAGs are not especially relevant when thinking about the positivity assumption, empirical strategies like regression discontinuity, or assumptions like monotonicity (Imbens, 2020; Pearl, 2010b). That is, neither approach is a silver bullet that can automatically solve causal inferential issues, but together they allow researchers to think through different assumptions and subtleties that are required to make causal claims.

## 5. Coda

In this paper, we brought to the fore some notions and empirical strategies that can help cultural evolutionary scholars tackle causal questions about culture with a richer toolbox. Rest assured, no solution is perfect, because causal inference, especially in the context of culture, is a complex endeavour. Yet a clear conceptual framework to think about causal effects, a combination of different designs and techniques, and a clear awareness of their strengths and limitations can provide the cultural evolutionary field with interesting opportunities when studying culture as cause.

## Data Availability

Data availability is not applicable to this article as no new data were created or analysed in this study.
